# ﻿*Stellariayabulaiensis* (Caryophyllaceae), a new species from Inner Mongolia, China

**DOI:** 10.3897/phytokeys.259.150512

**Published:** 2025-06-26

**Authors:** Lei Liu, Xiao-Jing Qiang, Fan Huang, Xiu-Zheng Huang, Ya-Lin Yu

**Affiliations:** 1 Grassland Research Institute of Chinese Academy of Agriculture Sciences, Hohhot 010010, China Grassland Research Institute of Chinese Academy of Agriculture Sciences Hohhot China

**Keywords:** Morphology, new species, *Stellaria*

## Abstract

*Stellariayabulaiensis* (Caryophyllaceae), a new species from Inner Mongolia, China, is described and depicted with photographic illustrations. It is similar to *S.bistyla*, from which differs in its slender fibrous root, longer leaves and pedicel, occurrence of three styles, and more seeds. A morphological comparison among the new species and the similar ones is also provided.

## ﻿Introduction

*Stellaria* L. (1753: 421) (Caryophyllaceae Juss.) is a genus containing 150–200 species occurring almost throughout the world (Hernández-Ledesma et al. 2015; POWO 2025). *Stellaria* is polyphyletic and many taxa have been transferred to other genera, like *Adenonema* Bunge (1836: 548), *Cerastium*Linnaeus (1753: 438), *Engellaria*Iamonico (2021: 4), *Hartmaniella* M.L.Zhang & Rabeler (2017: 455), *Mesostemma* Vvedensky (1941: 4), *Maguirellaria*Iamonico (2023: 240), *Minuartia* Linneaus (1753:89), *Mononeuria*Reichenbach (1845: 205), *Nubelaria* M.T.Sharples & E.A.Tripp (2019a: 886), *Pseudocherleria* Dillenberger & Kadereit (2014: 451), and *Rabelera* M.T.Sharples & E.A.Tripp (2019b: 870). After these changes, according to Sharples and Tripp (2019b) and Iamonico (2021, 2023), *Stellaria* would now consist of 111 species. A worldwide revision of *Stellaria* is still lacking (El Mokni et al. 2023).

More than seventy *Stellaria* species are currently recorded in China, including some newly described in recent years (Gan and Li 2014; Xu and Ma 2018; Song et al. 2020; Wang et al.2020, 2024; Yahara et al. 2021; Li et al. 2022; Arya et al. 2024). 27 species have been reported in Inner Mongolia according to Zhao et al. (2019).

As a key taxonomic feature, the majority of *Stellaria* species have three styles, but a notable variation is observed in certain species in which the number of styles ranges from two to four, as seen in *S.bistyla* Y.Z.Zhao, *S.alsine* Grimm, *S.vestita* Kurz, *S.graminea* L., *S.ovatifolia* (M.Mizush.) M.Mizush., *S.strongylosepala* Hand.-Mazz., etc.

In order to investigate the florist diversity of Alashan region (western Inner Mongolia, China), we observed a population of *Stellaria* which cannot be ascribed to any known species. After a careful examination of Floras and other relevant literature (Ren and Di 1985; Zhao YZ 1985; Lu et al. 2001) and exsiccate preserved in numerous herbarium, we reached the conclusion that a new species can be proposed.

## ﻿Materials and methods

Field surveys were carried out during the years 2023 and 2024. Morphological characters of living plants, including flowering and non-flowering individuals, were observed, measured and photographed under an SLR camera and an Olympus stereozoom microscope (OLYMPUS SZX10) and described using the terminology used by Lu et al. (2001). *Stellariabistyla* and *S.alsine* were considered for the morphological comparison according to Zhao et al. (2019). Specimens were deposited in the Herbarium FGC (acronym according to Thiers 2025). Further material is consulted through JSTOR Global Plants (2025), NSII (2025), and NPSRC (2025). Relevant literature is also analyzed (Lepsí et al. 2019; Sindhu and Singh 2024).

## ﻿Taxonomic treatment

### ﻿ Stellaria yabulaiensis

Taxon classificationPlantaeCaryophyllalesCaryophyllaceae

L.Liu sp. nov.

82B9EDBE-2367-5945-AB28-A4DB3E0A97F0

urn:lsid:ipni.org:names:77364104-1

Figs 1 , 2

#### Type.

China. Inner Mongolia: Alashan, Alashan Right Banner, Yabulai Town, 40°9'21"N, 104°0'12"E, 1367 m s.l.m., 8 August 2024, L.Liu *GRI2023102* (holotype: FGC!, isotype: FGC!).

#### Diagnosis

**(Table 1).***Stellariayabulaiensis* is similar to *S.bistyla* from which differ in having a slender fibrous root (0.3–0.5 mm in diameter *vs.* ca. 5–8 mm taproot), occurrence of a rhizome (*vs.* without rhizome), quadrangular stem (*vs.* cylindrical stem), linear-lanceolate leaf, 30–65 × 4–6 mm (*vs.* ovate-lanceolate, 10–20 × 2–10 mm), longer pedicel, 14–40 mm (*vs.* 3–20 mm), 3 styles (*vs.* usually 2 styles), and 1–3 seeds per fruit (*vs.*1–2 seeds).

**Table 1.** Morphological comparison amongst *Stellariayabulaiensis*, *S.bistyla*, and *S.alsine*.

		*S.yabulaiensis*	*S.bistyla*	*S.alsine*
**Plant height (cm)**		8–20	10–40	15–25
**Hair on the plant**		glandular hairy	glandular hairy	glabrous
**Root**		fibrous root slender	taproot (5–8 mm)	fibrous Root,slender
**Rhizome**		Yes	Yes	No
**Stem**		quadrangular	cylindicalr	quadrangular
**Leaf**	**Shape**	linear-lanceolate	ovate-lanceolate	lanceolate to oblong-lanceolate
	**Size**	30–65 × 4–6 mm	10–20 × 2–10 mm	5–20 × 2–4 mm
**Pedicel (mm)**		14–40	3–20	5–20
**Sepal**		ca.6 × 2 mm	4–5 × ca. 1 mm	2–4 × ca. 1 mm
**Petal (length)**		5.8–6.1 mm	ca. 3 mm	2–4 mm
**Capsule (length)**		2.3–2.7 mm	ca. 2.5 mm	subequaling or slightly longer than sepal
Number of styles		2 (3, obvious)	2 (3, rare)	3 (sometimes 2)
Seed number		1–3	1 (or 2)	More

#### Description.

Herbs perennial, 8–20 cm tall. Slender fibrous root. ***Rhizome*** quadrangular and slender (0.7–1.1 mm in diameter). ***Stems***, quadrangular, slender with glandular hairs. ***Leaves*** sessile, smooth, oblong to oblong-lanceolate, 3.0–6.5 cm long, 4–6 mm wide, apex acuminate, base slightly narrowed, margin entire, mid-vein obviously raised. ***Flowers*** solitary in axillary cymes. ***Bracts*** foliate, oblong, 3–15 mm long, 3 mm wide, apex acuminate. ***Pedicel*** filiform, glandular hairs, 1.4–4.0 cm. ***Sepals*** 5, glandular hairs, oblong, 6 mm long, 2 mm wide, margin membranous. ***Petals*** 5, subequalling sepals, 2-lobed cleft, lobes oblong. ***Stamens*** 10, almost equal with sepals, filament widened at base, succulent, anthers fuchsia. ***Ovary*** ellipsoid, styles 2 or 3. ***Capsule*** black-brown, ellipsoid, markedly shorter than sepals, ca. 2.5 mm long. ***Seeds*** 1–3, brown, reniform, 2 mm long, 1.5 mm wide, regularly rugulose.

**Figure 1.**
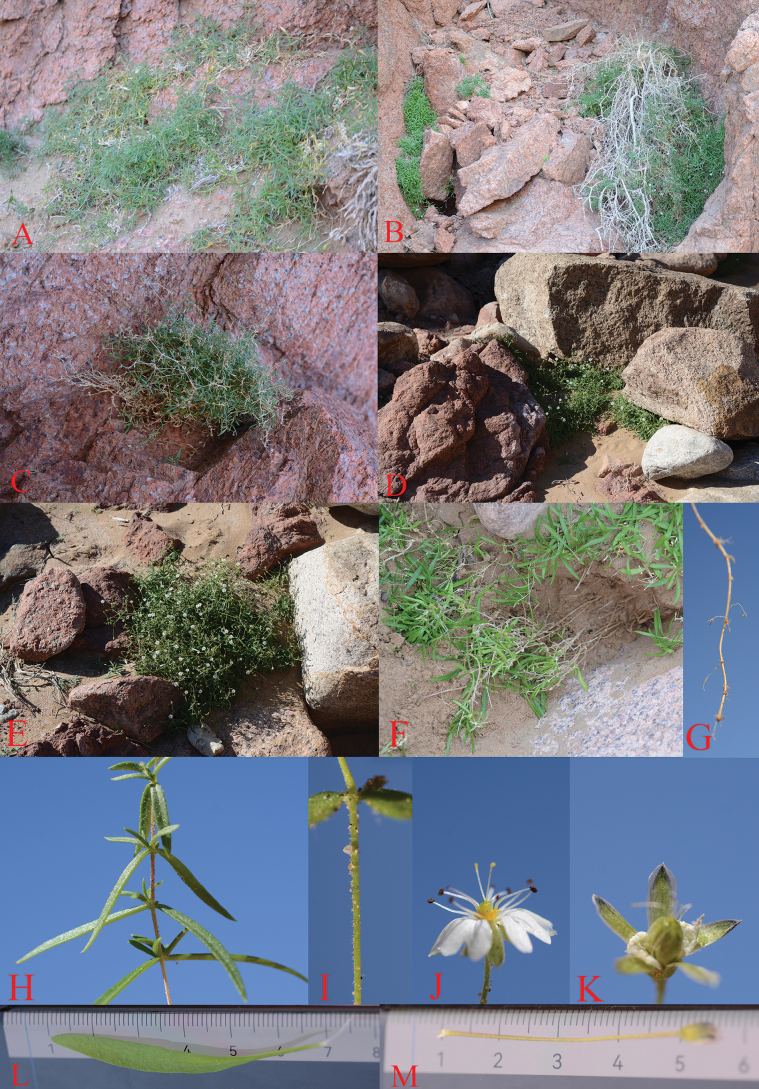
*Stellariayabulaiensis* L.Liu. **A–D.** Habitat; **E.** Plant; F Root; **G.** Rhizome; **H.** Branch; **I.** Stem (glandular hairs); **J.** Flower (style 2); **K.** Capsule; **L.** Leaf; **M.** Pedicel.

#### Etymology.

The specific epithet “yabulaiensis” refers to the Yabulai Mountain where the new species was collected.

#### Vernacular name

**(Chinese).** yǎ bù lài fán lǚ (雅布赖繁缕).

#### Distribution and habitat.

*Stellariayabulaiensis* is known from Alashan Right Banner, Inner Mongolia, China. The plants grow in humid sand at 1370 m a.s.l. For the time being, *S.yabulaiensis* is only known from the type locality, comprising less than 50 individuals.

**Figure 2.**
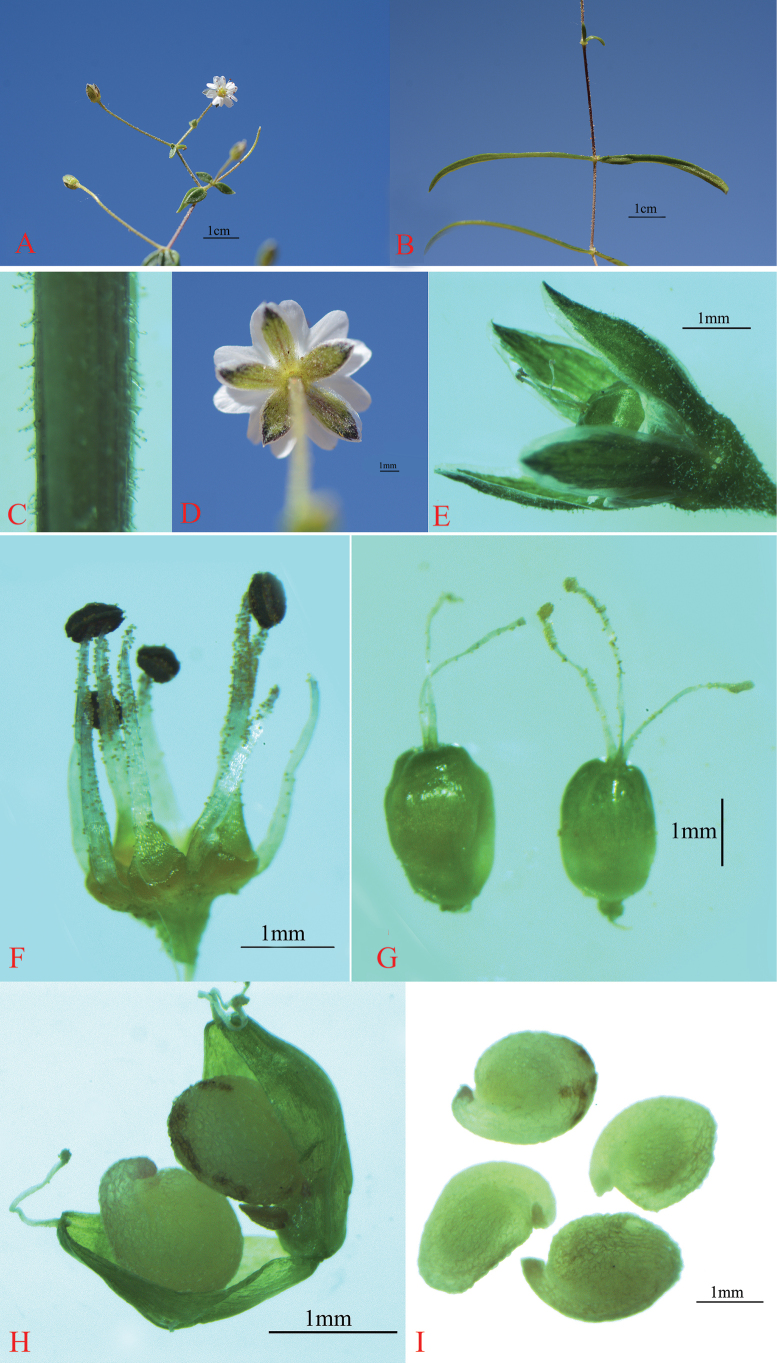
*Stellariayabulaiensis* L.Liu. **A.** Inflorescence; **B.** Leaf; **C.** Stem (glandular hairs); **D.** Flower (glandular hairs); **E.** Sepal; **F.** Stamen; **G.** Ovary (styles 2, 3); **H.** Capsule; **I.** Seeds.

#### Phenology.

Flowering time June-July; fruiting time August-October.

#### Conservation status.

*Stellariayabulaiensis* was found only in the type locality whose population are very small, but the whole distribution area is not clear; hence, we cannot conclude if it is a relatively common or rare species. In consideration of the dry area in western Inner Mongoilia, the new species needs further investigation in its distribution area and size of population to determine the current IUCN category.

#### Taxonomic notes.

Due to the large number of species within the genus *Stellaria*, it has been further classified into six sections: Sect. Leucostemma, Sect. Fimbripetalum, Sect. Adenonema, Sect. Stellaria, Sect. Oligosperma, and Sect. Schizothecium ; subsections and series occur within Sect. Stellaria. *Stellariayabulaiensis* belongs to Sect. Oligosperma since it displays mainly 2 styles, 5 petals, 5 sepals, and 10 stamens.

## Supplementary Material

XML Treatment for Stellaria yabulaiensis

## ﻿Additional information

### Data availability

All of the data that support the findings of this study are available in the main text.

## References

[B1] Arya S Kumar VNSA Sojan J Philip AA Suresh V (2024) *Stellariamcclintockiae* (Caryophyllaceae) a new species from Kerala, India. Phytotaxa 645(1): 55–65. 10.11646/phytotaxa.645.1.5

[B2] Bunge, Alexander von (1836) Mémoires de l’Académie impériale des sciences de St. Pétersbourg. 5e série. St.-Pétersbourg Divers Savans 2: 548.

[B3] China National Specimen Information Infrastructure [NSII] (2025) China National Specimen Information Infrastructure. http://www.nsii.org.cn/2017/query.php?name=\u7e41\u7f15\u5c5e [accessed 22 May 2025]

[B4] Chinese Virtual Herbarium [NPSRC] (2025) Chinese Virtual Herbarium. https://www.cvh.ac.cn/ [accessed 22 May 2025]

[B5] Dillenberger MS Kadereit JW (2014) Corrigendum to “Maximum polyphyly: Multiple origins and delimitation with plesiomorphic characters require a new circumscription of Minuartia (Caryophyllaceae)”. Taxon 63(2): 451–451. [Taxon 63: 64–88, S1–S21. 2014] 10.12705/632.42

[B6] El Mokni R Del Guacchio E Iamonico D (2023) Further insights into the *Stellariamediaaggregate* (Caryophyllaceae, Alsinoideae, Alsineae) in Africa: First reports of *S.ruderalis* in North Africa and *S.cupaniana* in Tunisia, with nomenclatural notes on the name *Alsinecupaniana*. Phytotaxa 584(4): 264–274. 10.11646/phytotaxa.584.4.3

[B7] Gan QL Li XW (2014) *Stellariazhuxiensis* (Caryophyllaceae), a New Species from Hubei, China. Annales Botanici Fennici 51: 22–24. 10.5735/085.051.0102

[B8] Hernández-Ledesma P Berendsohn WG Borsch T Mering SV Akhani H Arias S Castañeda-Noa I Eggli U Eriksson R Flores-Olvera H Fuentes-Bazán S Kadereit G Klak C Korotkova N Nyffeler R Ocampo G Ochoterena H Oxelman B Rabeler RK Sanchez A Schlumpberger BO Uotila P (2015) A taxonomic backbone for the global synthesis of species diversity in the angiosperm order Caryophyllales. Willdenowia 45: 281–383. 10.3372/wi.45.45301

[B9] Iamonico D (2021) *Engellaria* (Caryophyllaceae), a new North American genus segregated from *Stellaria*. Acta Botánica Mexicana 128: e1846. 10.21829/abm128.2021.1846

[B10] Iamonico D (2023) *Maguirellaria* (Caryophyllaceae), a new genus from Dominican Republic. Phytotaxa 598(3): 237–244. 10.11646/phytotaxa.598.3.5

[B11] JSTOR Global Plants (2025) JSTOR Global Plants. https://plants.jstor.org [accessed 22 May 2025]

[B12] Lepsí M Lepsí P Koutecky P Lucanová M Koutecká E Kaplan Z (2019) *Stellariaruderalis*, a new species in the *Stellariamedia* group from central Europe. Preslia 91(4): 391–420. https://doi 10.23855/preslia.2019.391

[B13] Li M Tian CF Song YF Ju WB Pathak ML Xu B (2022) *Stellariamotuoensis* (Caryophyllaceae), a new species from Xizang, China. Nordic Journal of Botany 03683. 10.1111/njb.03683

[B14] Linnaeus C (1753) Species Plantarum. Imprensis Laurentii Salvii, Holmiae, 89, 421, 438.

[B15] Lu DQ Wu ZY Zhou LH Chen SL Michael GG Magnus L John M John KM Bengt O Richard KR Mats T Nicholas JT Warren LW (2001) Caryophyllaceae. Flora of China 6. Sci. Press, Beijing & Missouri Bot. Garden Press, St. Louis, 1–113.

[B16] POWO (2025) Plant of the World Online. https://powo.science.kew.org/ [accessed 22 May 2025]

[B17] Reichenbach (1845) Deutsche Botaniker Erster Band. Das Herbarienbuch: Erklärung des natürlichen Pflanzensystems, systematische Aufzählung, Synonymik und Register der bis jetzt bekannten Pflanzengattungen. In der Arnoldischen Buchh, Dresden, 205 pp.

[B18] Ren Y Di WZ (1985) A new species of *Stellaria* from Helanshan Montain. Acta Botanica Boreali-Occidentalia Sinica 5(3): 231–232.

[B19] Sharples MT Tripp EA (2019a) Taxonomic observations within *Stellaria* (Caryophyllaceae): Insights from ecology, geofraphy, morphology and phylogeny suggest widespread parallelism in starworts and erode previous infrageneric classifications. Systematic Botany 44: 887–886. 10.1600/036364419X15710776741459

[B20] Sharples MT Tripp EA (2019b) Phylogenetic relationships within and delimitation of the cosmopolitan flowering plant genus *Stellaria* L. (Caryophyllaceae): Core stars and fallen stars. Systematic Botany 44(4): 857–876. 10.1600/036364419X15710776741440

[B21] Sindhu A Singh H (2024) A new species of *Stellaria* (Caryophyllaceae, Alsinoideae, Alsineae) from Eastern Himalaya, India. Phytotaxa 668: 177–185. 10.11646/phytotaxa.383.1.2

[B22] Song YF Li M Xu B Chen SF Chen L Xie CP (2020) *Stellariamultipartita* (Caryophyllaceae), a new species from Chongqing, China. Phytotaxa 442(3): 196–204. 10.11646/phytotaxa.442.3.5

[B23] Thiers B (2025) Index Herbariorum: A global directory of public herbaria and associated staff. New York Botanical Garden’s Virtual Herbarium. https://sweetgum.nybg.org/ [accessed 22 May 2025]

[B24] Vvedensky Alexei Ivanovich (1941) Botanicheskie Materialy Gerbariya Glavnogo Botanicheskogo. Uzbekistanskogo Filiala Akademii Nauk S.S.S.R. 3: 4.

[B25] Wang WQ Su ZW Ma ZH (2020) Lectotypification of five names in the genus *Stellaria* (Caryophyllaceae) in China. PhytoKeys 170: 71–81. 10.3897/phytokeys.175.59527 33442324 PMC7769891

[B26] Wang WQ Su ZW Ma ZH (2024) Phylogenetic and taxonomic studies of six recently-described *Stellaria* species (Caryophyllaceae) from China, with an additional new species, *Stellarialongipedicellata*, from Sichuan. PhytoKeys 249: 287–298. 10.3897/phytokeys.249.136456 39679360 PMC11638712

[B27] Xu HF Ma ZH (2018) *Stellariaabaensis* (Caryophyllaceae), a new species from China. Phytotaxa 383: 348–354. 10.11646/phytotaxa.383.1.2

[B28] Yahara T Hirota SK Fuse K Sato H Tagane S Suyama Y (2021) A new subspecies of *Stellariaalsine* (Caryophyllaceae) from Yakushima, Japan. PhytoKeys 187: 177–188. 10.3897/phytokeys.187.64023 35068974 PMC8738627

[B29] Zhang ML Zeng XQ Li C Sanderson SC Byalt VV Lei Y (2017) Molecular phylogenetic analysis and character evolution in Pseudostellaria (Caryophyllaceae) and description of a new genus, Hartmaniella, in North America. Botanical Journal of the Linnean Society 184(4): 444–456. 10.1093/botlinnean/box036

[B30] Zhao YZ (1985) The study of phytotaxonomy of the genus Stellaria in InnerMongolia. Bulletin of Botanical Research 5(4): 139–150.

[B31] Zhao YZ Zhao LQ Cao R (2019) Flora IntraMongolica (Ed3) Vol. 2. Inner Mongolia Renming Press, Hohhot, 16–35.

